# 1855. Understanding Cytomegalovirus (CMV) Symptom Management, Quality of Life, and Care Coordination in Transplant Recipients Through Patient and Care Partner (CP) Experiences

**DOI:** 10.1093/ofid/ofad500.1683

**Published:** 2023-11-27

**Authors:** Deepshikha Singh, Mary C Burke, Subhara Raveendran, Erlyn Rachelle Macarayan, Maisha Razzaque, Genovefa Papanicolaou, Macey L Levan, Maggie McCue, Megan Gower, Daniele K Gelone

**Affiliations:** PatientsLikeMe, Boston, Massachusetts; PatientsLikeMe, Boston, Massachusetts; PatientsLikeMe, Boston, Massachusetts; PatientsLikeMe, Boston, Massachusetts; PatientsLikeMe, Boston, Massachusetts; Memorial Sloan Kettering Cancer Center, New York, NY; NYU Grossman School of Medicine, New York, New York; Takeda Pharmaceuticals U.S.A., Inc., Lexington, Massachusetts; Takeda Pharmaceuticals U.S.A., Inc., Lexington, Massachusetts; Takeda Pharmaceuticals U.S.A., Inc., Lexington, Massachusetts

## Abstract

**Background:**

Transplant recipients with CMV infection have a high risk of complications and mortality. This study aimed to better understand patient and CP knowledge of post-transplant CMV and identify how it can be improved.

**Methods:**

The study included 2 research phases (**Fig 1**). We report results from Phase 2 (September 2022–March 2023), which included an educational webinar with a pre- and post-webinar assessment comprising 4 questions on the risk, symptoms, treatment, and complications of CMV; participants (pts) self-reported their confidence rating pre- and post-webinar. Transplant recipients and CPs also completed a quantitative survey on CMV management and patient/CP burden. Pts were recruited via the PatientsLikeMe (PLM) website, email, or social media.

**Figure:**
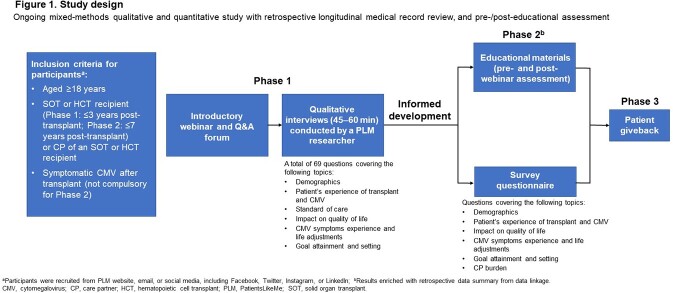

**Results:**

The webinar was completed by 33 pts. There was a total of 86 and 121 correct responses to the 4 pre- and post-survey questions, respectively, indicating a significant improvement in understanding of CMV post-webinar as compared with before (p< 0.05; t-test statistic). Almost half (n=16 [48%]) of pts reported feeling “not at all confident” in recognizing CMV symptoms post-transplant pre-webinar; post-webinar, this was 0 pts. Twenty-eight transplant recipients (27 solid organ transplant; 1 hematopoietic cell transplant) and 1 CP completed the quantitative survey. Half of recipients received a CMV diagnosis post-transplant and 16/28 reported having no awareness of their or their donor’s CMV status prior to transplant. Most (27/28) recipients reported a positive experience with their care team post-transplant. Half of recipients set goals with their care team or were provided resources to track them post-transplant. Recipients reported CMV medication changes and challenges with treatment (**Table 1**). Patients primarily contacted their surgeon about CMV and sought information on the internet.

**Figure:**
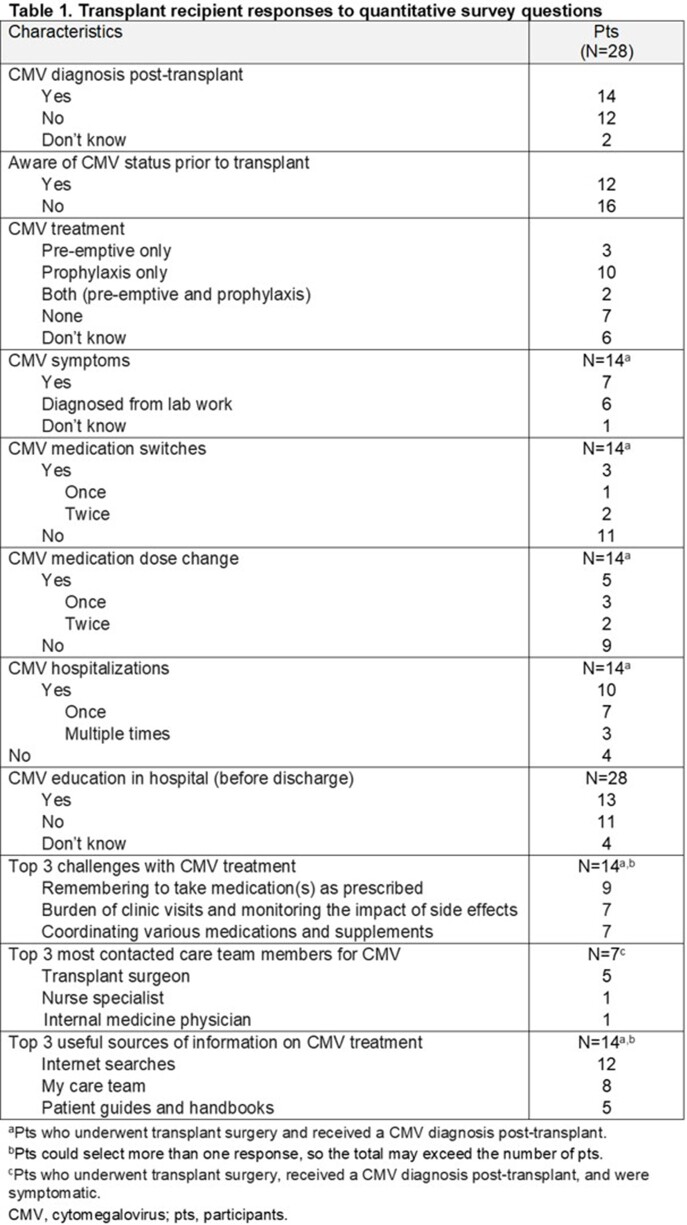

**Conclusion:**

Although transplant recipients reported positive experiences with their healthcare team, the post-transplant care journey could be improved by better educating patients about CMV and CMV management. Aggregate-level learnings from the study with be shared on the PLM platform to reinforce the value of participation in research.

**Funding:** Collaborative research study; primary funding from Takeda Pharmaceuticals U.S.A., Inc.

**Disclosures:**

**Deepshikha Singh, MPH**, PatientsLikeMe: Employee|PatientsLikeMe: Ownership Interest **Mary C. Burke, MHA**, PatientsLikeMe: Employee|PatientsLikeMe: Ownership Interest **Subhara Raveendran, PhD**, Ascendis Pharma: Employee|PatientsLikeMe: Ownership Interest **Erlyn Rachelle Macarayan, PhD**, PatientsLikeMe: Employee|PatientsLikeMe: Ownership Interest **Maisha Razzaque, MS**, PatientsLikeMe: Employee|PatientsLikeMe: Ownership Interest **Genovefa Papanicolaou, MD**, Allovir: Advisor/Consultant|Amplyx: Advisor/Consultant|Astellas: Advisor/Consultant|Cidara: Advisor/Consultant|CSL Behring: Advisor/Consultant|DSMC: Advisor/Consultant|Merck: Advisor/Consultant|Merck: Grant/Research Support|Merck: institutional research support for clinical trials|MSD: Advisor/Consultant|Octapharma: Advisor/Consultant|Partners Rx: Advisor/Consultant|Shire/Takeda: institutional research support for clinical trials|Symbio: Advisor/Consultant|Symbio: Advisor/Consultant|Takeda: Advisor/Consultant|Vera Pharma: Advisor/Consultant **Macey L. Levan, PhD**, Takeda Pharmaceuticals: Advisor/Consultant **Maggie McCue, MS, RD**, Takeda Pharmaceuticals U.S.A., Inc.: Employee **Megan Gower, PharmD**, Takeda Pharmaceuticals U.S.A., Inc.: Employee|Takeda Pharmaceuticals U.S.A., Inc.: Ownership Interest **Daniele K. Gelone, PharmD**, Abbott Laboratories: Ownership Interest|Ionis Pharmaceuticals: Ownership Interest|Johnson & Johnson: Ownership Interest|NovoNordisk: Ownership Interest|Pfizer: Ownership Interest|Regenxbio: Ownership Interest|Takeda Pharmaceuticals U.S.A., Inc.: Employee|Takeda Pharmaceuticals U.S.A., Inc.: Ownership Interest|Vertex Pharmaceuticals: Ownership Interest

